# Entropy Analysis of Soccer Dynamics

**DOI:** 10.3390/e21020187

**Published:** 2019-02-16

**Authors:** António M. Lopes, J. A. Tenreiro Machado

**Affiliations:** 1 UISPA–LAETA/INEGI, Faculty of Engineering, University of Porto, Rua Dr. Roberto Frias, 4200-465 Porto, Portugal; 2 Department of Electrical Engineering, Institute of Engineering, Polytechnic of Porto, Rua Dr. António Bernardino de Almeida, 431, 4249-015 Porto, Portugal

**Keywords:** entropy, mutual information, Jensen–Shannon divergence, fractional calculus, multidimensional scaling, complex systems

## Abstract

This paper adopts the information and fractional calculus tools for studying the dynamics of a national soccer league. A soccer league season is treated as a complex system (CS) with a state observable at discrete time instants, that is, at the time of rounds. The CS state, consisting of the goals scored by the teams, is processed by means of different tools, namely entropy, mutual information and Jensen–Shannon divergence. The CS behavior is visualized in 3-D maps generated by multidimensional scaling. The points on the maps represent rounds and their relative positioning allows for a direct interpretation of the results.

## 1. Introduction

Soccer (also known as association football, or football) is one of the most popular team sports around the world. It involves more than 250 million players in about 200 countries [1,2]. Five of the most prestigious national soccer leagues are located in Europe, namely the English “Premier League”, the Spanish “La Liga”, the German “Bundesliga”, the Italian “Serie A”, and the French “Ligue 1”. The total annual revenue of these leagues amounts to nearly 17 billion Euros. The game is played by two teams, composed of 11 players each, on a rectangular field with a goal placed at each end. The objective of the game is to score by getting a spherical ball into the opposing goal. The 10 field players can maneuver the ball using any part of the body except hands and arms, while the goalkeeper is allowed to touch the ball with the whole body, as long as he/she stays in his/her penalty area. Otherwise, the rules of the field players apply. The match has two periods of 45 min each. The winning team is the one that scores more goals by the end of the match.

For most European leagues, one season includes two parts, so that the visited and visitor teams interchange place. All teams start with zero points and, at every round one {victory, draw, defeat} worth {3,1,0} points. By the end of the last round, the team that accumulated more points is crowned champion.

A soccer league can be seen as a complex system (CS) constituted by multiple agents that interact at different scales in time and space. For example, at the match time scale, we observe interactions between players, coaches, referees, supporters, and environment, among others, that lead to a certain team performance during the match [1,3,4,5,6,7,8,9,10,11,12]. On the other hand, at the season time scale, we verify interactions between teams, at several matches, while the teams behavior evolves subject to transfers of players and coaches, injuries, suspensions, physical and mental stress, administrative decisions, and others [13,14,15]. Therefore, a plethora of elements gives rise to the emergence of a collective dynamics, with time-space patterns that can be analyzed by the mathematical and computational tools adopted for tackling dynamical systems [16,17].

Entropy-based techniques have been successfully applied in the study of many problems in sciences and engineering [18,19,20,21]. Divergence measures are tightly connected to entropy and assume a key role in theoretical and applied statistical inference and data processing problems, namely estimation, classification, detection, recognition, compression, indexation, diagnosis, and others [22]. We can mention the Kullback–Leibler [23], Hellinger [24], Csiszár α [22], Rényi [25], Jensen–Rényi [26,27], Jensen–Shannon [28] formulations, among others [22].

Fractional Calculus (FC) generalizes the classical differential operations to non-integer orders [29,30,31,32,33]. The area of FC dates back to the year 1695, in the follow-up of several letters between l’Hôpital and Leibniz about the meaning and apparent paradox of the *n*th-order derivative $\frac{d^{n}f\left(t\right)}{dt^{n}}$, for $n=\frac{1}{2}$. However, only in recent decades was FC recognized to play an important role in the modeling and control of many physical phenomena. FC emerged as a key tool in the area of dynamical systems with complex behavior and non-locality. Nowadays, the FC concepts are applied in different scientific fields, namely mathematics, physics, biology, finance and geophysics [34,35,36,37,38,39,40,41,42]. Indeed, fractional derivatives capture memory effects [43] and hereditary properties, providing a more insightful description of the phenomena [31,44,45,46].

In this paper, we adopt the information and FC theories for studying the evolution of a national soccer league season, while unveiling possible patterns in successive seasons. A soccer league season is treated as a CS with a state observable at discrete time instants, that is, at the time of rounds. The CS state consists of the goals scored by the teams, which is processed by means of different tools, namely entropy, mutual information and Jensen–Shannon divergence. The CS behavior is visualized using multidimensional scaling (MDS). The MDS generates maps of points in the 3-D space that represent the CS dynamics. The relative positioning of the points and the emerging patterns allow for a direct interpretation of the CS behavior. Therefore, with this scheme we can investigate the dynamics along each season, while embedding, indirectly, different time scales and entities at distinct levels of detail. In other words, we are not tackling a specific player, team, match, or entity, but the behavior of the CS that involves all aspects in a macro scale.

Bearing these ideas in mind, this paper is organized as follows. Section 2 describes the experimental dataset and includes a summary of the main characteristics and rules of each league. Section 3 introduces the main mathematical tools for processing the data. Section 4 analyses two top European soccer leagues. Finally, Section 5 outlines the main conclusions.

## 2. Dataset and Description of the Leagues

Data for worldwide soccer is available at http://www.worldfootball.net/. The database contains information about national leagues and international competitions. For the national leagues, the results of the matches are organized on a per season basis. For each match we know the names of the home and away teams, the goals scored, the points gathered, and the date of the match, along with other information.

We consider 23 seasons from the years 1995/1996 up to 2017/2018 of the two top national European leagues, namely the English “Premier League” and the Spanish “La Liga”. The “Premier League”, or “Premiership”, was established in 1992 as the most important league of the English association football. It involves 20 teams and adopts a system of promotion and relegation with the “Championship”, meaning that the three worst classified of the “Premier League” are relegated to the “Championship” and the three best classified of the “Championship” are promoted to the “Premier League”. The “Premier League” is now the most popular football league in the world, and the one that registers the highest stadium occupancy among all soccer leagues in Europe. “La Liga” started in 1929 as the top division of the Spanish soccer league system. It has been considered by UEFA the strongest league in Europe in recent years. Since 1997, “La Liga” engaged 20 teams. At the end of every season, the three lowest placed teams are relegated to the “Second Division”, and are replaced by the top two teams of this league plus the winner of a play-off competition.

## 3. Mathematical Fundamentals

This section introduces the main mathematical tools adopted for data processing, namely entropy, mutual information, Jensen–Shannon divergence and MDS.

### 3.1. Information Measures

Let us consider a discrete 1-D random variable *X* with possible values in $X=\{x_{1},\cdots ,x_{N}\}$ and probability mass function P(X). An event with probability of occurrence P(x), x∈X, has information content, *I*, given by:

(1) $$I\left[P\left(x\right)\right]=-\log P\left(x\right).$$

The expected value of *I* is the Shannon entropy:

(2) $$S\left(X\right)=E\left[-\log P\left(x\right)\right]=\sum_{x\in X}\left[-\log P\left(x\right)\right]P\left(x\right),$$

where $E\left(\cdot \right)$ denotes the expected value operator. Expression (2) satisfies the four Khinchin axioms [47,48] and measures the uncertainty in P(X).

The joint entropy quantifies the shape of the mass function associated with a set of random variables [49]. The joint Shannon entropy of two 2-D discrete random variables (X,Y) is defined as:

(3) $$S\left(X,Y\right)=\sum_{x\in X}\sum_{y\in Y}-P\left(x,y\right)\log P\left(x,y\right)=E_{X,Y}\left[I\left(X,Y\right)\right],$$

where P(X,Y) denotes the joint probability mass function. If *X* and *Y* are independent, then their joint entropy is the sum of the individual entropies, meaning that $S\left(X,Y\right)=S\left(X\right)+S\left(Y\right)$.

The mutual information MI is also an important concept in information theory. The MI quantifies the information shared by the two random variables. Loosely speaking, the MI measures the average amount of information in one random variable about the other. Formally, the MI of the random variables *X* and *Y*, with marginal probability mass functions P(X) and P(Y), respectively, is given by [50,51]:

(4) $$MI\left(X,Y\right)=\sum_{x\in X}\sum_{y\in Y}P\left(x,y\right)\log\frac{P\left(x,y\right)}{P(x)P(y)}=E_{X,Y}\left[SI\left(X,Y\right)\right],$$

where $SI\left(X,Y\right)=\log\frac{P\left(x,y\right)}{P(x)P(y)}$ denotes the pointwise mutual information.

When the random variables *X* and *Y* are independent, there is no shared information between them, and the mutual information is MI(X,Y)=0.

The Jensen–Shannon divergence between the two probability mass functions P(X) and P(Y) is given by [52]:

(5) $$JSD\left[P(X)∥P(Y)\right]=\frac{1}{2}\left[\sum_{x\in X}P\left(x\right)\ln P\left(x\right)+\sum_{y\in Y}P\left(y\right)\ln P\left(y\right)\right]-\sum_{z\in Z}P\left(z\right)\ln P\left(z\right),$$

where $P\left(z\right)=\frac{P(x)+P(y)}{2}$.

### 3.2. A Fractional Calculus Approach to Information Measures

In the scope of the Shannon approach, we note that the information, $I\left[P\left(x\right)\right]=-\log P\left(x\right)$, is a function between the cases $D^{-1}I\left[P\left(x\right)\right]=P\left(x\right)\left[1-\log P\left(x\right)\right]$ and $D^{1}I\left[P(x)\right]=-\frac{1}{P(x)}$, where *I* and *D* denote the integral and derivative operators, respectively. The interpretation of these expressions in the perspective of FC led to rewriting the information and entropy of order α∈R as follows [53,54]:

(6) $$I_{\alpha }\left[P\left(x\right)\right]=D_{0^{+}}^{\alpha }I\left[P\left(x\right)\right]=-\frac{P{\left(x\right)}^{-\alpha }}{\Gamma \left(\alpha +1\right)}\left[\log P\left(x\right)+\psi \left(1\right)-\psi \left(1-\alpha \right)\right],$$

(7) $$S_{\alpha }\left(X\right)=\sum_{x\in X}\sum_{y\in Y}\left\{-\frac{P{\left(x\right)}^{-\alpha }}{\Gamma \left(\alpha +1\right)}\left[\log P\left(x\right)+\psi \left(1\right)-\psi \left(1-\alpha \right)\right]\right\}P\left(x\right),$$

where $\Gamma \left(\cdot \right)$ and $\psi \left(\cdot \right)$ represent the gamma and digamma functions, respectively.

Similarly, for the pair of random variables *X* and *Y* the joint fractional entropy of order α∈R can be written as:

(8) $$S_{\alpha }\left(X,Y\right)=\sum_{x\in X}\sum_{y\in Y}\left\{-\frac{P{\left(x,y\right)}^{-\alpha }}{\Gamma \left(\alpha +1\right)}\left[\log P\left(x,y\right)+\psi \left(1\right)-\psi \left(1-\alpha \right)\right]\right\}P\left(x,y\right).$$

Expressions (6)–(8) lead to the Shannon information and entropies when α=0.

The fractional mutual information results in:

(9) $$MI_{\alpha }\left(X,Y\right)=\sum_{x\in X}\sum_{y\in Y}\left\{\frac{{\left[\frac{P(x,y)}{P(x)P(y)}\right]}^{-\alpha }}{\Gamma \left(\alpha +1\right)}\left[\log\frac{P(x,y)}{P(x)P(y)}+\psi \left(1\right)-\psi \left(1-\alpha \right)\right]\right\}P\left(x,y\right).$$

Using Equations (5) and (6), leads to the fractional (generalized) Jensen–Shannon divergence:

(10) $$\begin{array}{cc} JSD_{\alpha }\left[P(X)∥P(Y)\right]= & \frac{1}{2}\sum_{x\in X}P\left(x\right)\left\{\frac{P{\left(x\right)}^{-\alpha }}{\Gamma \left(\alpha +1\right)}\left[\ln P\left(x\right)+\psi \left(1\right)-\psi \left(1-\alpha \right)\right]\right\}+\\ & \frac{1}{2}\sum_{y\in Y}P\left(y\right)\left\{\frac{P{\left(y\right)}^{-\alpha }}{\Gamma \left(\alpha +1\right)}\left[\ln P\left(y\right)+\psi \left(1\right)-\psi \left(1-\alpha \right)\right]\right\}-\\ & \sum_{z\in Z}P\left(z\right)\left\{\frac{P{\left(z\right)}^{-\alpha }}{\Gamma \left(\alpha +1\right)}\left[\ln P\left(z\right)+\psi \left(1\right)-\psi \left(1-\alpha \right)\right]\right\}. \end{array}$$

For α=0 expression (10) reduces to the JSD defined in (5).

### 3.3. Multidimensional Scaling

MDS is a computational technique for clustering and visualizing data [55]. In a first phase, given *W* items in a *c*-dim space and a measure of dissimilarity, we calculate a W×W symmetric matrix, $\Delta =[\delta_{rs}]$, r,s=1,⋯,W, of item to item dissimilarities. The matrix Δ represents the input of the MDS numerical scheme. The MDS rational is to assign points for representing items in a *d*-dim space, with d<c, and to reproduce the measured dissimilarities, $\delta_{rs}$. In a second phase, the MDS evaluates different configurations for maximizing some fitness functions, arriving at a set of point coordinates (and, therefore, to a symmetric matrix of distances $\Phi =[\phi_{rs}]$ that represents the reproduced dissimilarities) that best approximates the original $\delta_{rs}$. A fitness function used often is the raw stress:

(11) $$S={\left[\phi_{rs}-f\left(\delta_{rs}\right)\right]}^{2},$$

where f(·) indicates some type of transformation.

The MDS interpretation is based on the patterns of points visualized in the generated map. Similar (dissimilar) objects are represented by points that are close to (far from) each other. Thus, the information retrieval is not based on the point coordinates, or the geometrical form of the clusters, and we can rotate, translate and magnify the map, since the distances remain identical. The MDS axes have neither units nor special meaning.

The quality of the MDS is evaluated by means of the stress and Shepard plots. The stress plot represents the locus of S versus the number of dimensions *d*. The map is a monotonic decreasing chart and choosing the value of *d* is a compromise between achieving low values of S or *d*. Often we adopt the values d=2 or d=3, since they allow a direct visualization. The Shepard diagram, for a particular value of *d*, compares $\phi_{rs}$ and $\delta_{rs}$. A narrow scatter around the 45 degree line represents a good fit between $\phi_{rs}$ and $\delta_{rs}$.

## 4. Analysis and Visualization of Soccer Data

### 4.1. Analysis of Soccer Data Based on Information Measures

The top European leagues engage *M* teams that play R=2(M−1) rounds. Throughout one season, each team has R/2 matches at home and R/2 matches away from home. For the *m*th team, m=1,⋯,M, at round *r*, r=1,⋯,R, we define the variables:

- $g_{m}\left(r\right)$—goals scored at home;
- $h_{m}\left(r\right)$—goals scored at home of the adversary.

For a league season the data is processed by means of the following steps:

1. define a M×M dimensional matrix, *A*, initialized with void elements;
2. at the end of round r=1,⋯,R update $A\left(r\right)=[a_{ij}\left(r\right)]$ such that $a_{ij}\left(r\right)\leftarrow g_{m}\left(r\right)$ and $a_{ji}\left(r\right)\leftarrow h_{m}\left(r\right)$. Therefore, at each round, *r*, a total of M/2 cells of A(r) are updated with new information based on the results of the matches;
3. normalize the matrix A(r) by calculating $\hat{A}\left(r\right)=\left[{\hat{a}}_{ij}\left(r\right)\right]$, where:
  (12) $${\hat{a}}_{ij}\left(r\right)=\frac{a_{ij}\left(r\right)}{\sum_{i=1}^{M}\sum_{j=1}^{M}a_{ij}\left(r\right)};$$
4. interpret $\hat{A}\left(r\right)$ as a 2-D probability mass function, and calculate the information measures S(r), $S_{\alpha }\left(r\right)$, IM(r) and $IM_{\alpha }\left(r\right)$.

**Figure 1:**
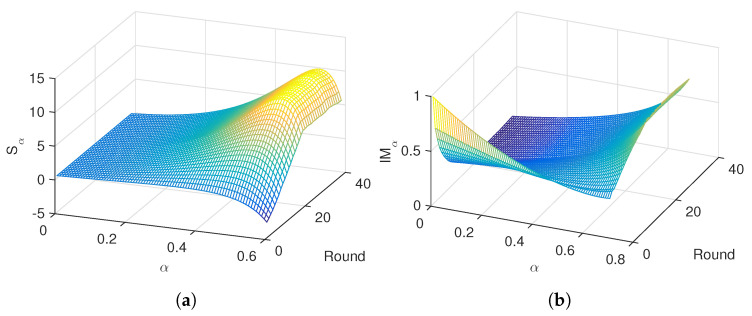
Evolution of (**a**) $S_{\alpha }$ and (**b**) $IM_{\alpha }$ versus α∈[0,0.6] and round, r=1,⋯,38, for the ”Premier League“ in season 2014/2015.

In the follow-up we apply the proposed numerical scheme. The order α=0.5 was adjusted experimentally as a compromise between maximizing sensitivity of the information measures and smoothing the transient at the beginning of the curves. Figure 1 illustrates the variation of $S_{\alpha }$ and $IM_{\alpha }$ versus α∈[0,0.6] and round, r=1,⋯,38, for the “Premier League” in season 2014/2015. We verify that close to α=0.5 the entropy, $S_{\alpha }$, is maximum, while for both $S_{\alpha }$ and mutual information, $IM_{\alpha }$, the transients are smooth. For other seasons and leagues we obtain the same type of results.

**Figure 2:**
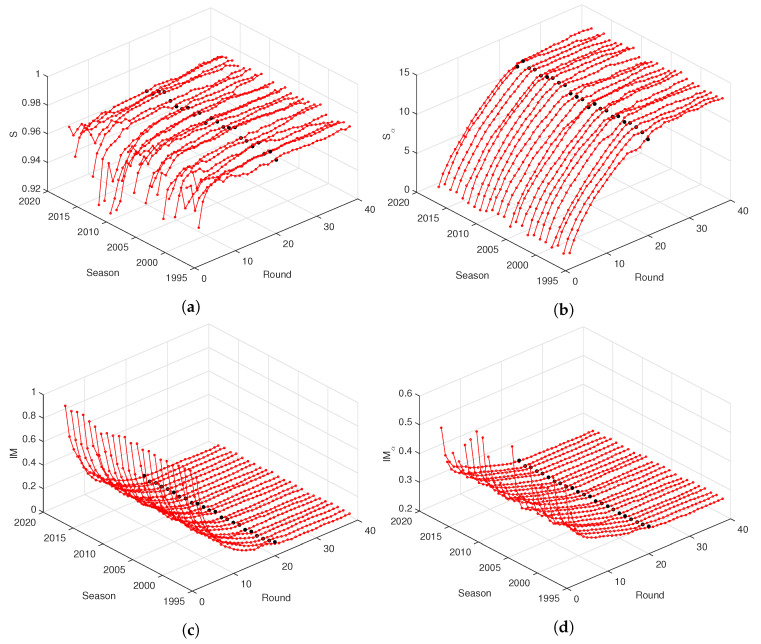
The entropy, (**a**) *S* and (**b**) $S_{\alpha }$, and mutual information, (**c**) IM and (**d**) $IM_{\alpha }$, with α=0.5, versus round, *r*, for the “Premier League” during the seasons 1995/1996 up to 2017/2018. The black marks denote half season r=19.

**Figure 3:**
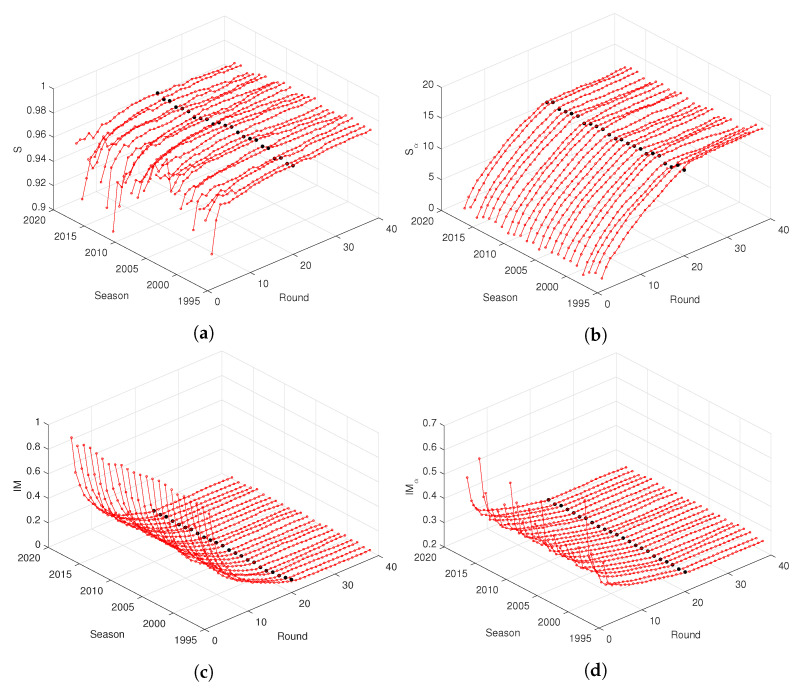
The entropy, (**a**) *S* and (**b**) $S_{\alpha }$, and mutual information, (**c**) IM and (**d**) $IM_{\alpha }$, with α=0.5, versus round, *r*, for the “La Liga” during seasons 1995/1996 up to 2017/2018. The black marks denote half season r=19.

Figure 2 and Figure 3 depict the entropy, *S* and $S_{\alpha }$, and mutual information, IM and $IM_{\alpha }$, versus round, r=1,⋯,38, for the “Premier League” and “La Liga”, during the seasons from 1995/1996 up to 2017/2018. For other national leagues, the graphs are of the same type. We verify the emergence of similar patterns on the charts both for *S* and $S_{\alpha }$, and IM and $IM_{\alpha }$, independently of the season. In fact, after an initial transient due to data scarcity and period of adaptation of the teams, visible mainly in *S*, all information measures evolve smoothly with *r*. The entropy increases and the mutual information decreases in time, while we observe that the fractional measures are less noisy.

For each league and season the information measures S(r), $S{\left(r\right)}_{\alpha }$, IM(r) and $IM{\left(r\right)}_{\alpha }$ are approximated by the function:

(13) $$f\left(r\right)=a\cdot b^{r}\cdot r^{c},\;a,b,c\in R.$$

**Figure 4:**
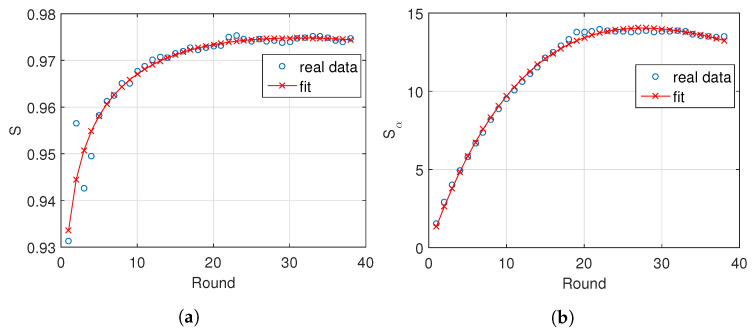

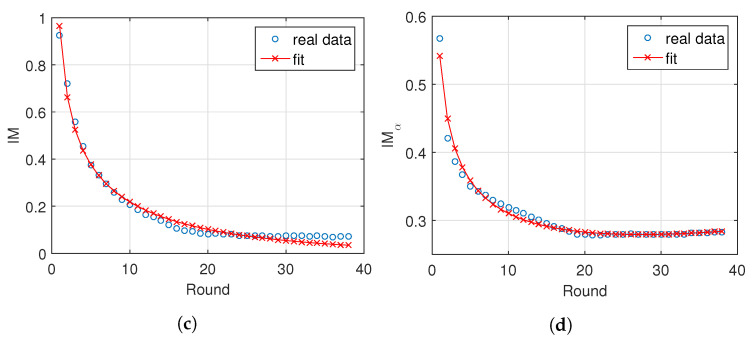
The $f\left(r\right)=a\cdot b^{r}\cdot r^{c}$ fit of the information measures, (**a**) *S*, (**b**) $S_{\alpha }$, (**c**) IM and (**d**) $IM_{\alpha }$, with α=0.5, for the “Premier League” in season 2014/2015.

**Figure 5:**
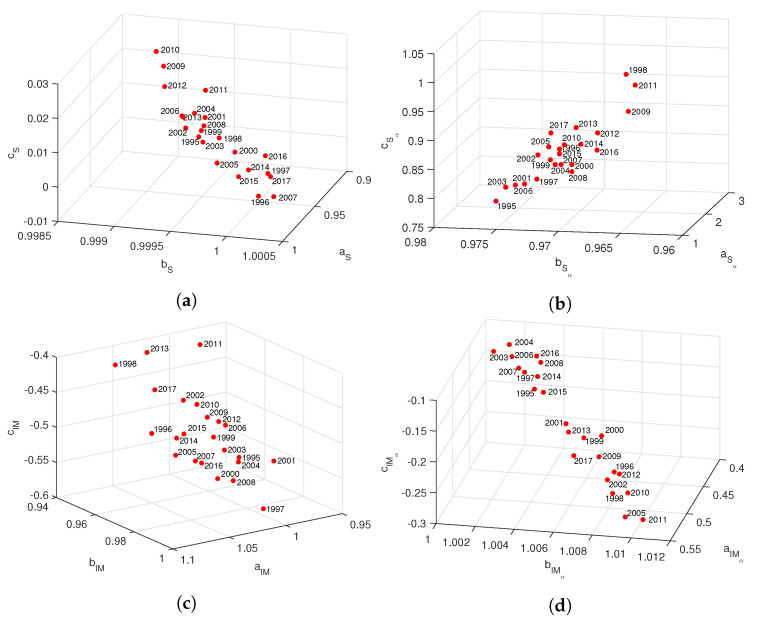
Locii of the parameters of $f\left(r\right)=a\cdot b^{r}\cdot r^{c}$ for the information measures, (**a**) *S*, (**b**) $S_{\alpha }$, (**c**) IM and (**d**) $IM_{\alpha }$, with α=0.5, for the “Premier League” in seasons 1995/1996 to 2017/2018.

Several numerical experiments demonstrated that model (13) fits well to the data and has a reduced number of parameters. Figure 4 illustrates the fit of *S*, $S_{\alpha }$, IM and $IM_{\alpha }$, with α=0.5, for the “Premier League” in season 2014/2015. Figure 5 depicts the locii (a,b,c) for seasons 1995/1996 to 2017/2018, where we observe a strong correlation between the three parameters. On the other hand, comparing the time evolution we find a strong variation, due to the known volatility of the results.

**Figure 6:**
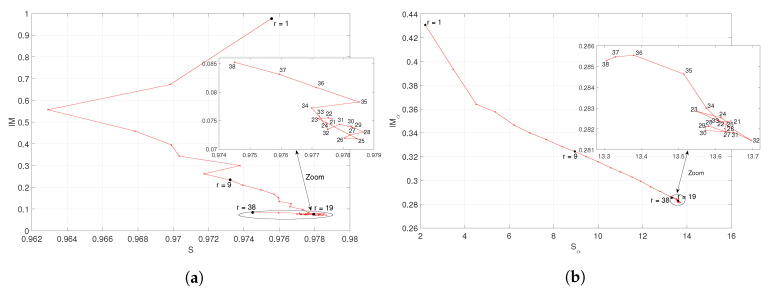
Locii of (**a**) (IM,S) and (**b**) $(IM_{\alpha },S_{\alpha }$, α=0.5), respectively, for the “Premier League” in season 2014/2015.

**Figure 7:**
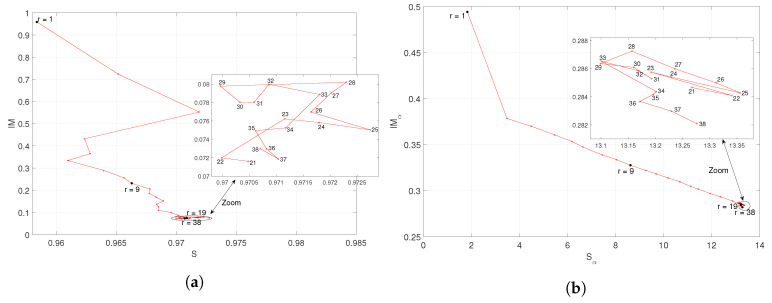
Locii of (**a**) (IM,S) and (**b**) $(IM_{\alpha },S_{\alpha }$, α=0.5), respectively, for the “La Liga” in season 2014/2015.

Inspired by the phase plane technique, widely used in the analysis of dynamical systems [42,56], we interpret the entropy and mutual information as phase variables of a soccer league season and we analyze the phase plane trajectories. Figure 6 and Figure 7 depict the locii of IM versus *S* and $IM_{\alpha }$ versus $S_{\alpha }$, respectively, for the “Premier League” and “La Liga” in season 2014/2015. For other seasons, the locii are of the same type. We note that after an initial transient, the locci converge smoothly towards a given region of the plane. In the final part, the trajectories unveil a complex behavior. We conjecture that such patterns are due to the proximity of the end of the season. In fact, a few rounds before the end of the season, we verify that some teams play demotivated, since they have already been relegated to the lower division, or they have already achieved their objectives. Conversely, other teams compete with extra motivation, since they are close to achieving their main endeavor.

### 4.2. Clustering and Visualization of Soccer Data Based on Information Measures

In this subsection we adopt the Jensen–Shannon divergence and the MDS technique to study the dynamics of a soccer league season. The MDS input are the dissimilarity matrices:

(14) $$\Delta =[JSD\left(\hat{A}\left(r\right)∥\hat{A}\left(s\right)\right)],\;r,s=1,\cdots ,R,$$

(15) $$\Delta_{\alpha }=\left[JSD_{\alpha }\left(\hat{A}\left(r\right)∥\hat{A}\left(s\right)\right)\right],\;r,s=1,\cdots ,R.$$

**Figure 8:**
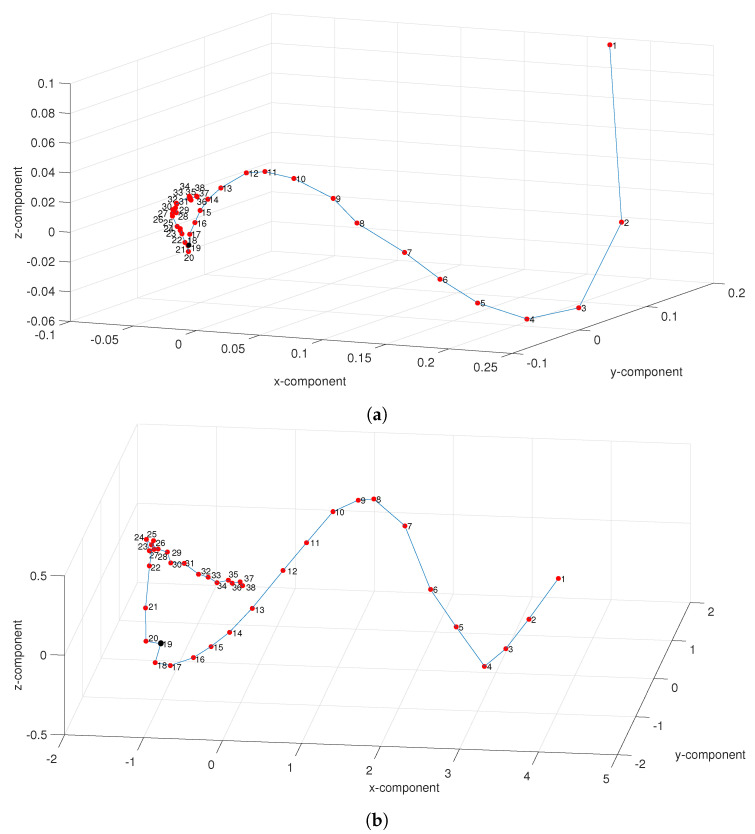
The 3-D MDS map based on matrices (**a**) Δ (14) and (**b**) $\Delta_{\alpha }$ (15), with α=0.5, for the “Premier League” in season 2014/2015. The black mark denotes half season r=19.

Figure 8 depicts the 3-D MDS maps (i.e., d=3) obtained with the dissimilarity matrices Δ (14) and $\Delta_{\alpha }$ (15) for the “Premier League” in season 2014/2015. For other seasons the results are of the same type. On both maps we observe the time-flow captured by the relative position of the points that represent rounds. For $\Delta_{\alpha }$ the evolution is more clear, namely in the second part of the league, revealing the superiority of the fractional information measures. Nonetheless, we find a considerable volatility between the distinct seasons, confirming the previous plots of the parameters (a,b,c).

**Figure 9:**
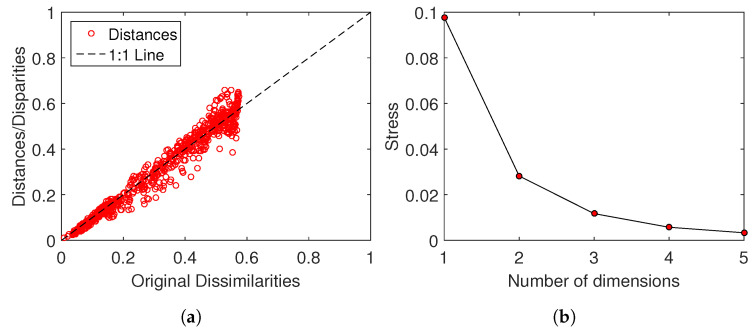
(**a**) Shepard and (**b**) stress plots assessing the quality of the MDS with matrix Δ for the “Premier League” in season 2014/2015.

Figure 9 shows the Shepard and stress plots that assess the MDS results obtained with the distance matrix Δ for the “Premier League” in season 2014/2015. The Shepard diagram shows that the points are distributed around the 45 degree line, indicating a good fit between the original and the reproduced distances. The stress plot reveals that a 3-D space (d=3) describes well the locus of points, since for d=3 we obtain the maximum curvature of the line and, therefore, 3-dim maps represent a good compromise between accuracy and visualization performance. For the matrix Δ the Shepard and stress plots lead to similar conclusions.

**Figure 10:**
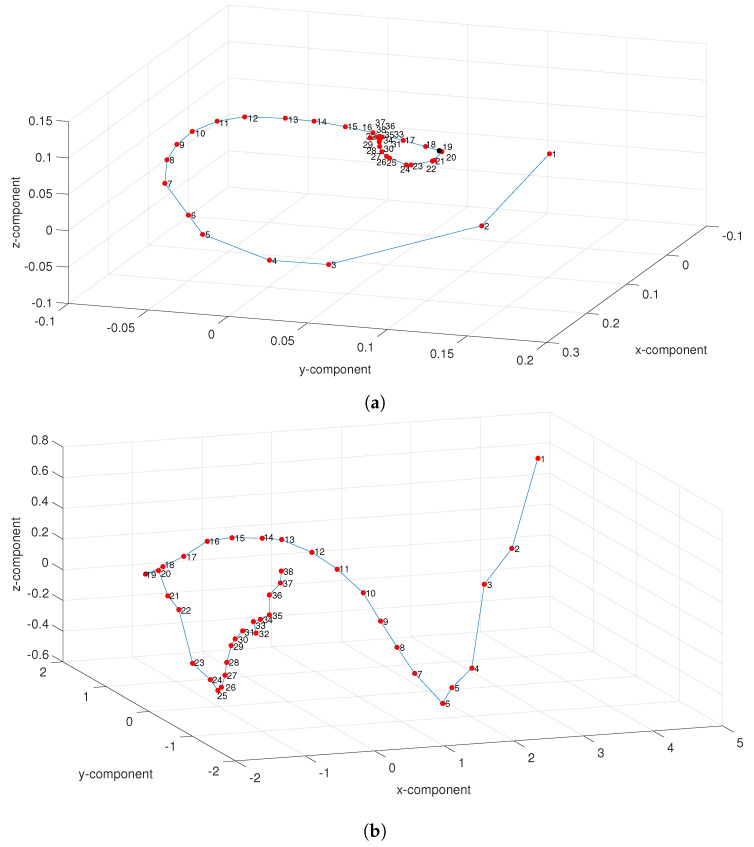
The 3-D MDS map based on matrices (**a**) Δ (14) and (**b**) $\Delta_{\alpha }$ (15), with α=0.5, for the “La Liga” in season 2014/2015. The black marks denote half season r=19.

Figure 10 shows the 3-D MDS maps obtained with the dissimilarity matrices Δ (14) and $\Delta_{\alpha }$ (15) for the “La Liga” in season 2014/2015. The results are consistent with those obtained for the “Premier League”, demonstrating that both leagues have the same type of dynamics when viewed in the perspective of the adopted information measures.

The applied ideas and tools were applied to a non-standard field, yielding realistic results. We do not expect that they are directly applicable in the field, but we believe that they will trigger future developments.

## 5. Conclusions

We proposed an original scheme based on information and fractional calculus tools for studying the dynamics of a national soccer league. A soccer league season was treated as a CS that evolves in discrete time, i.e., the rounds time. We considered that the CS state consisted of the goals scored by the teams, and we processed it by means of different tools, namely entropy, mutual information and Jensen–Shannon divergence. The CS behavior was visualized in 3-D maps generated by MDS. Fractional-order information measures were accurate in describing the complex behavior of such challenging systems.

The area of application is not classic and present-day tools adopted in that area are very limited. The proposed idea follows the perspective of applied mathematics, computer science and physics. It is not intended to develop a product directly applicable in the field. The authors believe that this initial study and others to follow will trigger future applications.
